# Early diagnosis of skin cancer by ultrasound frequency analysis

**DOI:** 10.1002/acm2.12671

**Published:** 2019-10-08

**Authors:** Shabnam Kia, Saeed Setayeshi, Majid Pouladian, Seyed Hossein Ardehali

**Affiliations:** ^1^ Department of Medical Radiation Engineering, science and research branch Islamic Azad University Tehran Iran; ^2^ Faculty of Energy Engineering and Physics Amirkabir University of Technology (Tehran Polytechnique) Tehran Iran; ^3^ Department of Biomedical Engineering, science and research branch Islamic Azad university Tehran Iran; ^4^ Research Center of Engineering in Medicine and Biology, Science and Research Branch Islamic Azad University Tehran Iran; ^5^ Department of Anesthesiology & Critical Care Shohadaye‐Tajrish hospital Shahid Beheshti University of Medical Sciences Tehran Iran

**Keywords:** biological resonance theory, discrete cosine transform, early detection, frequency analyzes, skin cancer diagnosis, skin sonography

## Abstract

The diagnosis of cancer by modern computer tools, at the very first stages of the incident, is a very important issue that has involved many researchers. In the meantime, skin cancer is a great deal of research because many people are involved with it. The purpose of this paper is to introduce an innovative method based on tissue frequency analyzes to obtain the accurate and real‐time evaluation of skin cancers. According to the Biological resonance theory, body cells have natural and unique frequencies based on their biological fluctuations, which, if the structure, profile and cellular status change, its frequency also varies. This concept and theory is considered as the basis for analyzing skin tissue health in the proposed method. Reflected ultrasound waves from tissue have been processed and studied based on frequency analysis as a new method for early detection and diagnosis of accurate location and type of skin diseases. The developed algorithm was approved through 400 patients from CRED; its ability to evaluate benign and malignant skin lesions was shown (AUC = 0.959), with comparable clinical precision; as for the selected threshold, sensitivity, and specificity were 93.8% and 97.3%, respectively. Therefore, this method can detect skin malignancy with an accurate, noninvasive and real‐time procedure.

## 1 INTRODUCTION

Currently detection of the malignancy of skin lesions, is doing by dermatologists based on their professional experience using the pathologic results from the skin biopsy of the suspicious area; and in some cases, meanwhile the skin biopsy of the suspected area, they also recommend the sonography imaging in order to inspect the skin tissue better and in more details.^1^ Sonography is a noninvasive method by which radiologists try to capture unusual symptoms in the skin sonograms.^2^ Due to the complication of sonograms in appearance, the diagnosis of malignant skin depends on the self‐experience of the dermatologist. This means that in most cases, the early symptoms of malignant lesions seems to be normal, and ignored.^2^ This causes the many false detection. Since these errors are always hazardous, there is significant interest in developing intelligent methods for detecting these abnormalities as a useful tool for dermatologists to accelerate the detection and prevention of unnecessary skin biopsy.^3^

The aim of this research is developing an intelligent diagnosis method for diagnosing malignant skin lesions, but the distinction between this work and previous researches is the new perspective on processing the sonograms based on frequency analyzes.

Biological resonance method is based on findings on biophysics and quantum mechanics. Quantum mechanics, in short, identified that everything in universe is a compressed energy and that each emits its own unique electromagnetic frequency. This means that all substance and therefore all cells, parts of body, etc. emit electromagnetic waves. Depending on their nature, all substances have a unique wavelength or frequency with highly individual characteristics. This is known as a frequency pattern.^4^

Thus any exchange of body nature oscillations that take place between the various cells in the body, can lead to detect an abnormality in that part of body.^4^

The main issue raised in this research is the use of the above concept to carry out the diagnostic process. In fact, both benign and malignant cells of skin are alive and have their natural oscillations that indicate their mode of life; based on research, the effect of nature oscillation frequency attend in ultrasonic echoes received from one part of the skin tissue, so they can be extracted and identified by using frequency processing and applied to classify these cells.

Two major tools utilized in this research are discrete cosine transform (DCT) and Otsu's thresholding method. DCT transform as the frequency analyzer and Otsu's method as the thresholder for distinguishing healthy and suspicious tissues from each other in a single sonogram. Both are widely used in biomedical image processing. DCT transform is used for different applications such as water marking,^18^, ^19^ compression,^20^, ^21^ and classification.^22^, ^23^ For example, Gutta and Cheng in their work used DCT of an autocorrelation function for biometric recognition using ECG signals.^37^ Also, in the field of cancer prediction and diagnosis (concerning what we are doing here), Lahmiri and Boukadoum have investigated DCT and Radon Transforms (RT) as a feature extraction tool in order to perform mammogram classification based on Support Vector Machine (SVM).^38^ These researches support the idea that cosine transform is promising as a feature. Otsu's method is also used for different purposes including thresholding^29^, ^30^ and segmentation.^31^ For instance, Lahmiri in his work^39^ has tried to outperform Otsu's method by improved variants of particle swarm optimization (PSO) algorithms in segmentation of biomedical images, namely brain, breast and prostate tissues. This shows the stability of this method and its promising results, although it could be replaced by more improved methods. Moreover, George in their work^40^ has used Otsu's method as a means for elimination of false‐positive (FP) findings (noisy circles and blood cells) in the cytological images of breast cancer. Using Otsu's thresholding, c‐means clustering technique and different topologies of neural networks, they have developed a remote computer‐aided breast cancer diagnosis system with a challenging performance.

According to the above, in brief, the conceptual principles of the study, the methods, and the results are described in this paper. Following the introduction to the first part, Part 1 examines background, Section 2 describes the methods of research, and in Section 3 the two algorithms proposed in this research are presented. Finally, in Section 4, the discussion and conclusion of the proposed methods are described.

## 2 BACKGROUND

Skin, the body's first defense system against invasive pathogens, is the largest and most prolific organism and has more than 16% of the body weight. Because of its importance, research on skin structure and its functionality has been so extensive that their achievements in the last decade are greater than those found over the last two centuries.^5^

### 2.1 Skin cancer

Skin cancer is one of the most common cancers in the world. In fact, the number of skin cancers in the world that is diagnosed every year is more than the number of all other cancers. The number of cases of skin cancer has increased significantly over the last few decades.^6^

Depending on the type of cells eroded, there are several types of skin cancer that have certain symptoms. The most common types of skin cancer in the ascending order of harmful effects include basal cell carcinoma (BCC), squamous cell carcinoma (SCC), and melanoma.^2^

### 2.2 Current diagnosis methods

Until now, diagnosing of skin diseases has been performed by the specialist physician's self‐experience and results of pathological tests; meaning that in most cases, if the specialist figures out any sign of disease, the patient is directed to pathology laboratory to do skin biopsy. To increase diagnosing accuracy, some specialists prescribe imaging as well as biopsy process. As we know anatomically, skin is classified as soft tissues, so an appropriate imaging method is needed.^2^

Among all of the image acquisition methods, sonography is one of the appropriate methods. With the ability of feature extraction of skin tissues, it can not only separate the lesions from the healthy parts but also specification detection of the lesion is noticeable.^2^

**Figure 1:**
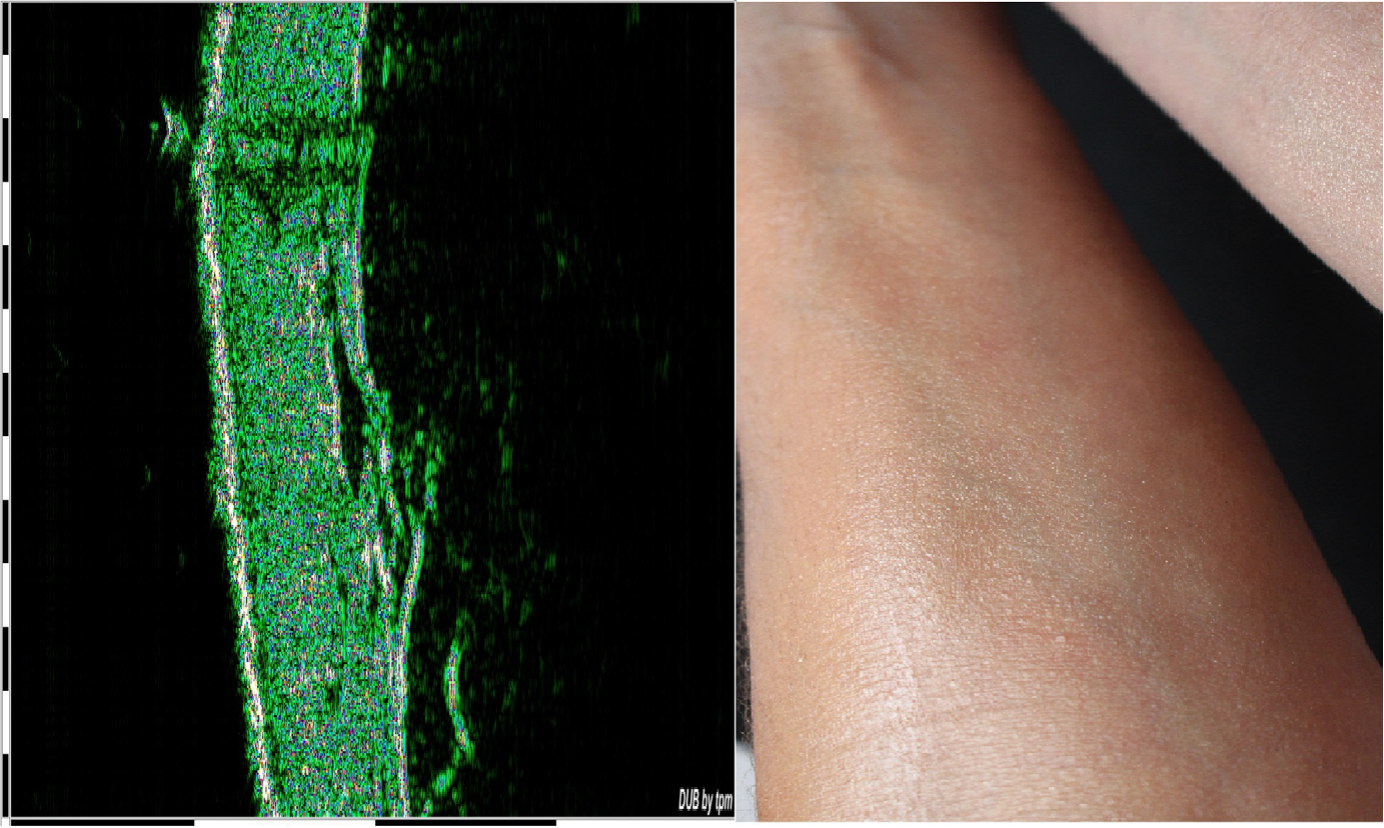
Sample of healthy skin and its sonogram.

The common frequency range for skin sonography is 20–100 MHz. There are two parameters for determining the range of frequency in ultrasound waves in sonography imaging system. First the depth of examining part of skin and second the desired resolution.^2^ Figure 1 shows the sample two‐dimensional (2D) sonogram of a normal skin with 14 × 7 mm dimensions. The length of the mentioned image shows the motion of the probe along the surface of the skin, and the width of the image shows the depth of the texture.^7^ In the normal skin epidermis, dermis and hypodermis sections are approximately alike and monotonous.^8^

**Figure 2:**
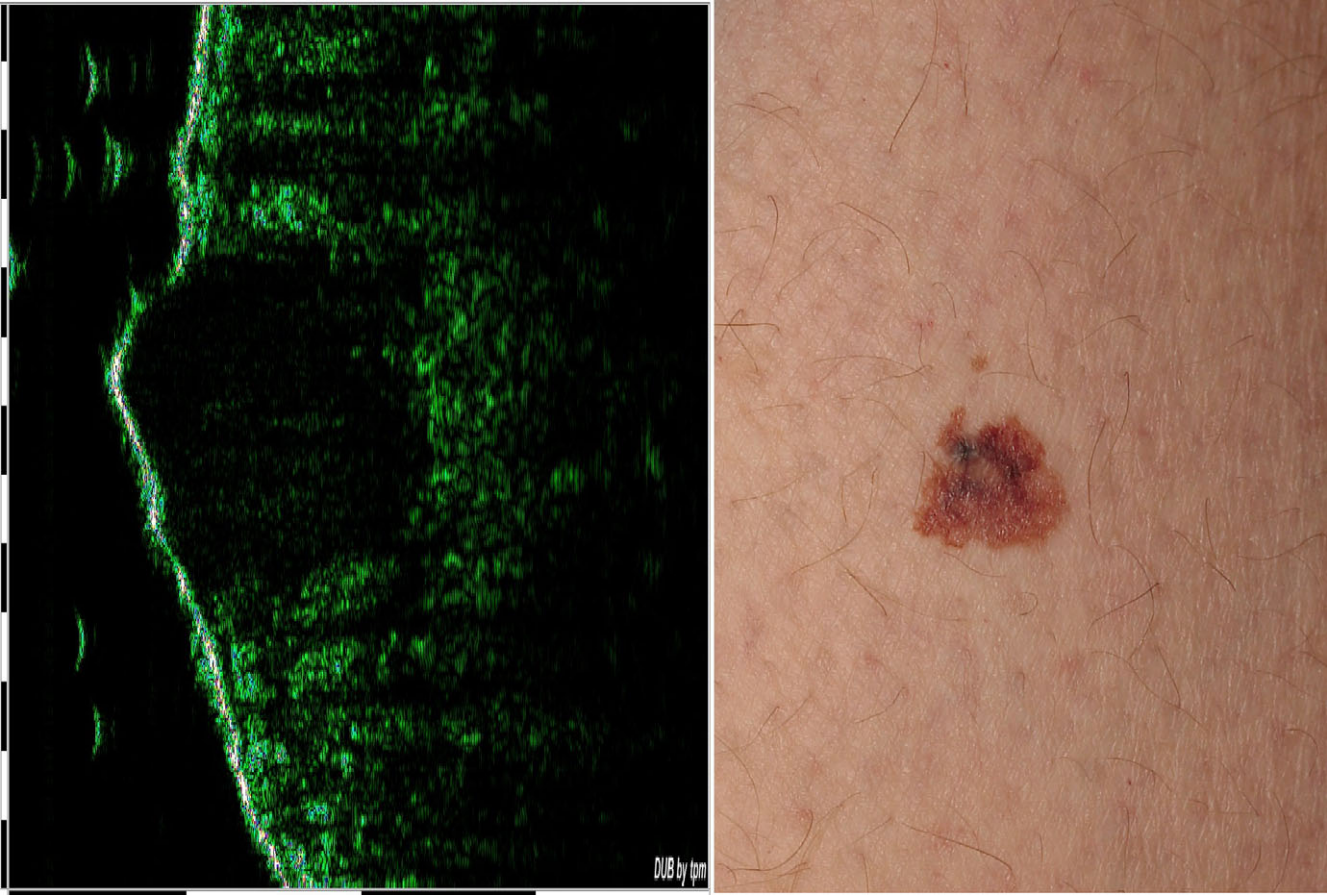
Sample of cancerous skin and its sonogram.

Because of the fat existence in these layers, these normal parts of skin seem brightly. On the other hand, the structure of malignant skin lesions with angiogenesis texture^9^ is not homogeneous; therefore, depending on the skin layer in which the lesion is located, there is a dark gap screened in the sonogram. These lesions have little ultrasonic echoes therefore they generally appear in dark colors in the sonograms (Fig. 2).^2^

### 2.3 The effect of tissue changes on sonograms frequency pattern

The main feature of this research is described in this section, which is the main difference with other related studies in this area. Here is focused on analyzing the frequency of the image that shows the structure of the skin tissue. Depending on the concept of biological resonance, corrupted body cells emit certain energy wavelengths that can be investigated.^10^ This theory states that each cell in the body has a specific frequency based on the current biological status. In another perspective, this idea can be considered correctly as the concept behind the strings^11^ in the so‐called M‐theory^12^ or the energy of the particle photon E with its associated ν frequency in the Planck‐Einstein relation (Eq. 1).

(1) E=h·v

where E = energy of the photon, h = Planck's constant, and ν stands for frequency.

It can also be deduced from this concept that cells in the body produce a certain frequency in the event of a disorder. Accordingly, and based on numerous studies in this field, it should be noted that so far no research has been done to analyze the effect of electromagnetic waves due to natural fluctuations in tissue (skin tissue) on the mechanical waveform recorded in the imaging system sonography. Needless to say that the purpose of this research is not to investigate electromagnetic waves or its frequency on the tissue cell. The purpose of this research is to make similar analyzes in the field of mechanical waves with the idea of Biological resonance. In other words, in this research the mechanical response of skin tissue analyzes to find a main feature for diagnosing the skin condition.

In this paper, the cells of the body are exposed to ultrasonic pulses. Since the wave length of these mechanical pulses is much smaller than the normal wavelength of the cells, therefore it can be considered relatively as an impulse in cells. Dirac delta input is a useful method to analyze systems' dynamics is the impulse response or the response of a system in control theory and signal processing. The dynamic system and its impulse response may be actual physical objects.^13^

The cells have an impulse as the input, thus stimulated to intensify their natural frequency; and this frequency will be in the content of each sample scan, by ultrasound device in this case, at that time.^14^, ^15^, ^16^

The sonography probe sends ultrasound waves and records the intensity of the pulse echo and reconstructs the 2D image. In reconstructing image procedure, the mentioned natural frequency from different parts of the skin tissue is mapped on the corresponding part of image and leads to the different echoes in the image. So by analyzing the frequency patterns of ultrasound image, a new golden biomarker to classify the different skin will be obtained. At last with extracting the special frequency features from both type of skin lesions (benign and malignant), these lesions can be classified with high precision.

## 3 METHODOLOGY

In the next sections, two algorithms are designed to perform the diagnosis procedure. But before discussing the algorithms we first need to enlighten some topics about our sonogram dataset, their format (RF and B‐Mode) and the transform we are using to take the sonograms into the frequency space.

### 3.1 Preparing dataset

Since the main objective of this study is to distinguish malignant skin lesions from benign, the analysis of the tissue requires a large amount of data, so that access to more information increases the accuracy of the system, thus requiring a number of many examples of ultrasound of various tissues in different conditions for processing are clearly visible.

The proposed algorithm was confirmed through a 2 yr database of 400 patients (aged 18–68 yr old) from the *Center of Research and Training in Skin Diseases*. The format and information of some of the used data are presented in Table 1.

**Table 1:** A few examples of data used in research.

Patient no.	Sample of malignant lesions data			Sample of benign lesions data		
	Age	Date of visit	Tumor dimensions (cm^2^)	Age	Date of visit	Tumor dimensions (cm^2^)
1	42	Feb‐08	1.05	57	May‐09	0.43
2	29	Jul‐14	1.70	23	Sep‐09	0.78
3	33	Jan‐14	1.98	46	Apr‐13	1.01
4	38	Oct‐13	0.70	75	Jul‐07	0.85
5	54	May‐07	1.75	69	Jan‐14	0.97
6	37	Jul‐03	1.06	34	May‐15	0.59
7	42	Mar‐15	1.50	45	Oct‐10	1.76
8	40	Sep‐11	0.76	63	Jul‐10	1.11
9	36	Aug‐04	1.30	28	Apr‐08	0.67
10	46	Aug‐14	1.70	71	Oct‐14	1.48
11	25	Jun‐14	0.85	49	Aug‐11	0.68
12	37	Nov‐11	1.10	36	Dec‐11	0.83
13	26	Jul‐11	1.80	29	Jun‐07	0.92
14	44	Sep‐08	1.06	49	Nov‐14	1.32
15	58	Apr‐07	2.34	64	May‐11	1.85

This vast database includes different types of skin lesions, such as melanomas, basal cell carcinomas, squamous cell carcinomas, actinic keratosis, atypical nevi, benign melanocytic nevi, blue nevi, and seborrheic keratosis. Of the 400 ultrasound samples used in this study, 220 cases are malignant, and the remaining lesions are benign. All of these samples were examined with pathologic results that were performed after scanning.

### 3.2 Preprocessing

The ultrasound scanner records data scans along with the patient's metadata in the binary file. The most important metadata in this study, other than image size, was the frequency of transducer. All samples of the ultrasound wave database are at a frequency of 50 MHz.

**Figure 3:**
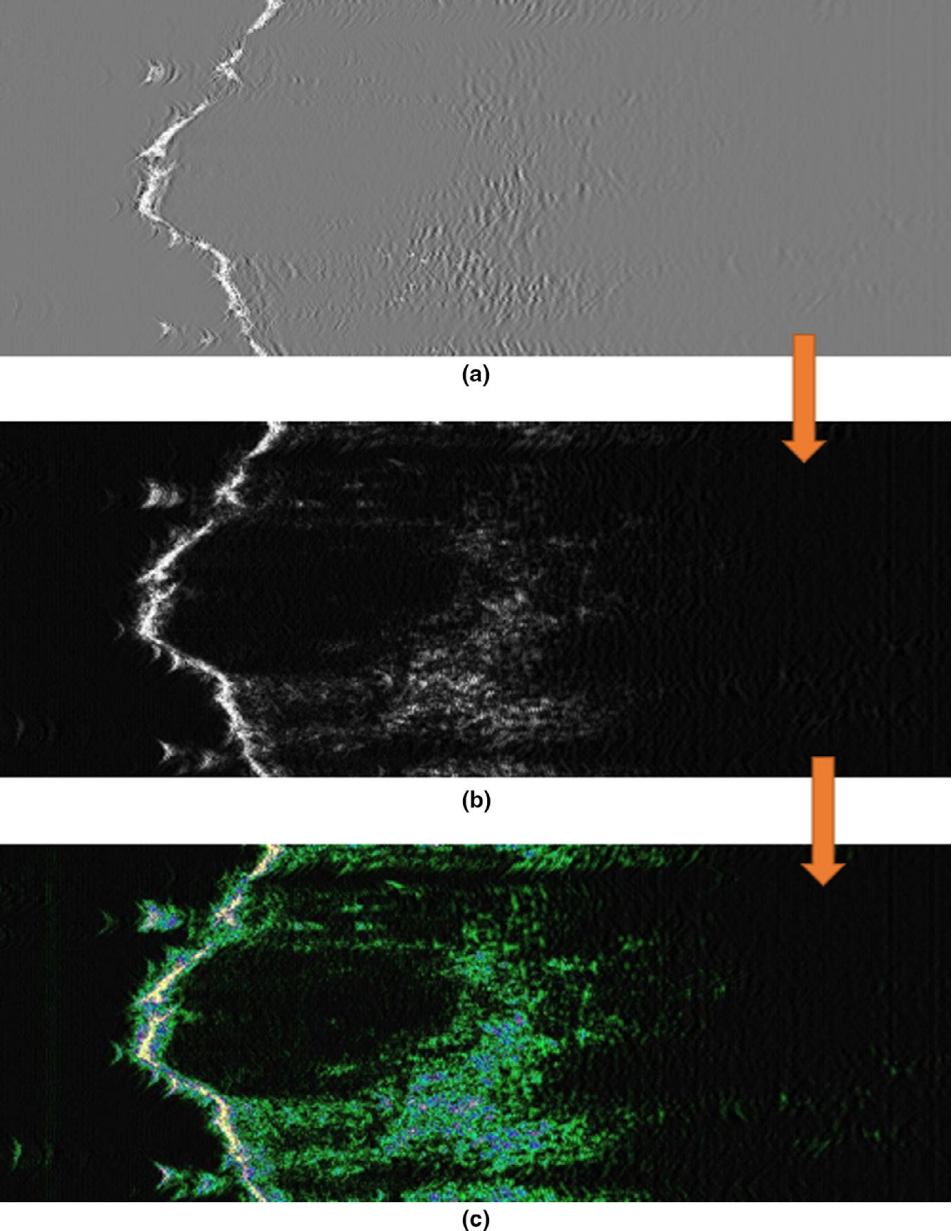
50 MHz sonogram used in this project (a) RF mode (b) B‐mode (c) customized color map.

Figure 3(a) shows the raw RF image. Then, the RF images turn to B‐Mode to be used in the next steps of this image instead of raw image. The goal is to enhance the quality of the images in order to be analyzed. Because the analysis and processing of images in the RF mode are hard and not precise. We use Eq. (2) to compute the B‐Image from the RF data:

(2) $$img_{B}=2\times abs\left(img_{\mathrm{RF}}\right)-offset$$

where offset would be a constant integer that the device uses in order to easily have all the recorded values as unsigned integers. Figure 3(b) shows main image that all calculations and trends apply. However, this is not visually familiar, so a custom color scheme has been applied to it, the result of which is shown in Fig. 3(c).

### 3.3 Frequency analysis

To frequency analysis of ultrasound imaging data from skin texture, the data must be transmitted to the frequency space. The most common frequency conversions used in image processing are fast fourier transform (FFT) and discrete cosine transforms (DCT).

A DCT shows a set of finite sequence of data points as complete cosine functions at different frequencies. These transformations are widely used in image processing. From compression, the loss of audio data, such as MP3s and images such as JPEGs, can be removed by small particles with high frequencies, to spectral methods for numerical solutions of the differential equation with partial derivatives in the range of DCTs.

Since less cosine functions are needed to approximate an ordinary signal (relative to sinusoidal functions), using the cosine function instead of sinus is necessary in compression. Also, in the case of differential functions, cosine functions have more specific boundary conditions.

Mainly, DCT is used for those processes in which low‐frequency content (such as nature frequencies of the body), should be considered. Nevertheless, the discrete fourier transform (DFT) offers a better means or intentions for spectral analysis, and the maps draw those results to very simple physical frequencies.^17^ The great advantage of DCT calculation is that it contains the required frequencies based on the size of the image, and the calculations will be meaningful and, accordingly, the DCT is chosen for this study. Mathematical expressions of DCT calculations of an M‐by‐N matrix “A” are presented in Eqs. (2), (3), and (4).

(3) $$B_{\mathrm{pq}}=\alpha_{p}\alpha_{q}\sum_{m=0}^{M-1}\sum_{n=0}^{N-1}A_{\mathrm{mn}}\cos\frac{\pi \left(2m+1\right)p}{2M}\cos\frac{\pi \left(2n+1\right)q}{2N},\begin{array}{c} 0\leq p\leq M-1\\ 0\leq q\leq N-1 \end{array}$$

(4) $$\begin{array}{c} \alpha_{p}=\left\{\begin{array}{c} \begin{array}{cc} \frac{1}{\sqrt{M}}, & p=0\\ \sqrt{\frac{2}{M},} & 1\leq p\leq M-1 \end{array} \end{array}\right. \end{array}$$

(5) $$\begin{array}{c} \alpha_{q}=\left\{\begin{array}{c} \begin{array}{cc} \frac{1}{N}, & q=0\\ \sqrt{\frac{2}{N},} & 1\leq q\leq N-1 \end{array} \end{array}\right. \end{array}$$

where $B_{\mathrm{pq}}$ are called the DCT coefficient of “A”.

DCT transform is widely used in biomedical image processing for different applications such as water marking,^18^, ^19^ compression,^20^, ^21^ and classification.^22^, ^23^

## 4 PROCEDURE

Here, we explain the diagnosis methods. The first method is used to classify the sonogram based on a frequency transform of the whole image, and the second method is used for sematic segmentation of the sonogram in order to differentiate the healthy parts from the suspected parts. We also have compared the first method to our previous work on the same dataset in terms of diagnosis quality and computation complexity and speed. The programming of different parts has been done in MATLAB, but it can be developed in any programming language.

### 4.1 Sonogram classification

**Figure 4:**
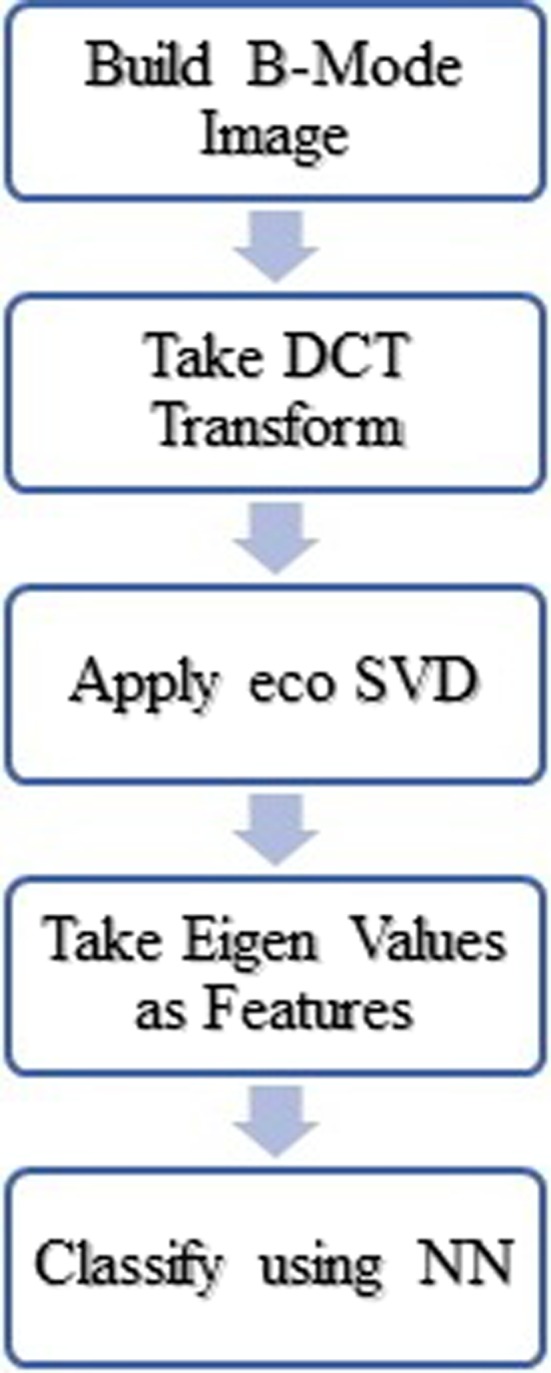
Procedure scheme for sonogram classification.

The first diagnosis method is used to classify the sonogram based on a frequency transform of the whole image. We also have compared this method to our previous work on the same dataset in terms of diagnosis quality and computation complexity and speed. The flowchart of the classification procedure is shown in Fig. 4. Each step is explained as following.

#### 4.1.1 Convert RF Image to B‐Mode

As mentioned before, first and foremost we need to do some preprocessing in order to build the B‐Mode image from the RF data. This is a critical step in the procedure since it is much easier to analyze the B‐Mode image than the raw RF input.

#### 4.1.2 Apply DCT transform

**Figure 5:**
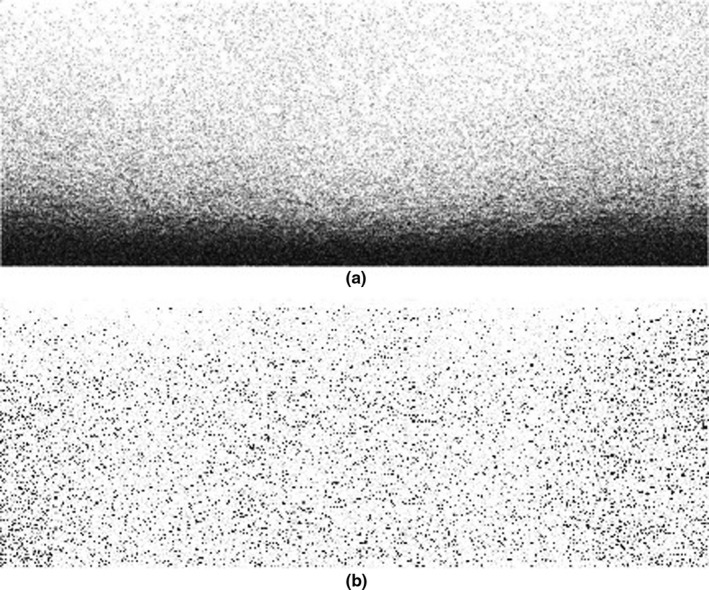
Frequency transform of (a) malignant lesion (b) benign lesion.

Figure 5 shows the absolute conversion of DCT for two different sonograms that one of them is nevus and the other has BCC. Based on the analysis carried out on the collected data, it is found that the frequency domain in the cosine transmission of malignant ultrasonography tissue is lower than that of benign and healthy tissues. This is a great way to find the lesions or infected parts of skin in the image. By measuring the amplitude and frequency, the size of the affected parts can also be calculated.

#### 4.1.3 Find Eigen values using SVD

When we calculate the DCT for the whole sonogram, we have a 2D matrix with the same dimensions of the original sonogram. We can use this image as the input vector for a deep network which is widely used nowadays, however, the computational resources for the deep‐learning approaches is much high and it would be better to avoid using them in case we can find a better solution. The dimension reduction algorithms such as principal component analysis (PCA) offer us a way to reduce the feature map size to a more affordable and concentrated values that have more eclectic covariance. A very powerful tool is singular value decomposition (SVD). SVD is a factorization of a real or complex matrix. It is the generalization of the Eigen decomposition of a positive semidefinite normal matrix to any matrix via an extension of the polar decomposition. SVD is extensively used in biomedical image processing algorithms as image compressor or feature extractor and so on.^24^, ^25^

In this research, we apply an economy‐size SVD as following equation:

(6) $$S=svd(log\left(abs\left(dct(image_{B-Mode}\right)\right)$$

**Figure 6:**
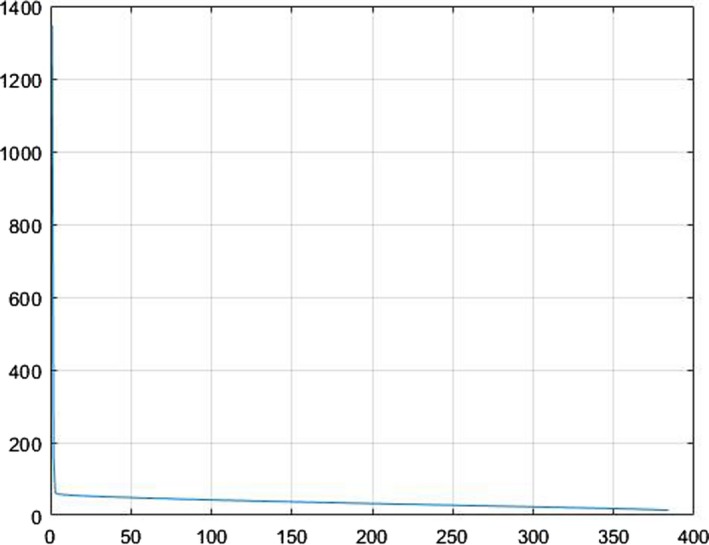
Plot of the singular value decomposition eigenvalues. DCT, discrete cosine transform.

A sample plot of the eigenvalues is shown in Fig. 6. We can see that the first eigenvalues have more energy than the other, but since in biomedical image processing, the high energy parts are mostly alike in different images and the differences lies within the low energy values, we keep all the values as the feature map for classification.

#### 4.1.4 Classify using neural network

**Figure 7:**
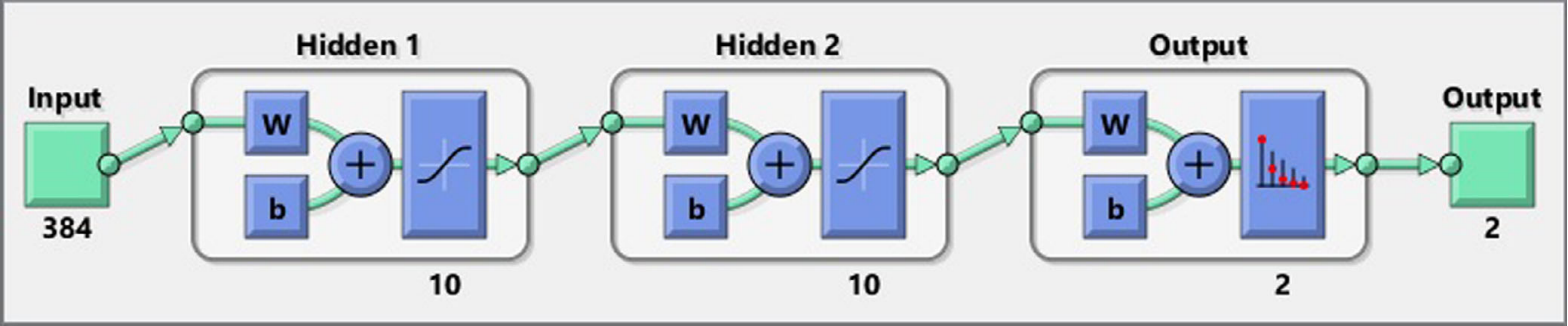
The pattern recognition network topology.

**Figure 8:**
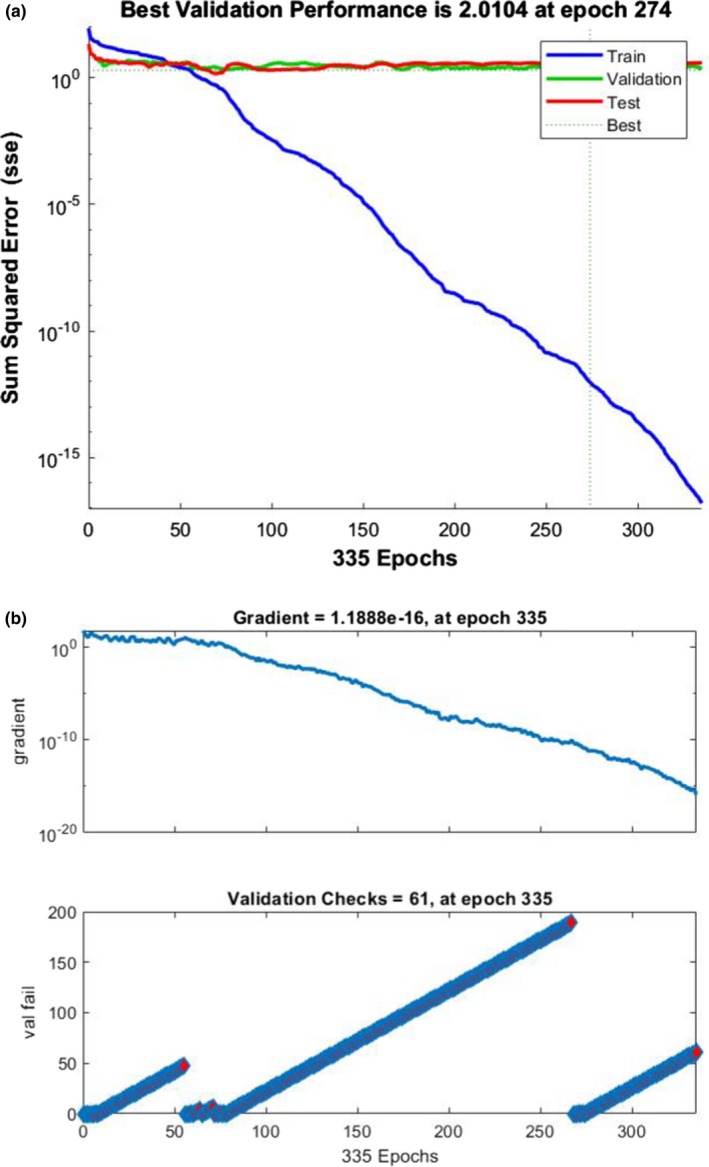
(a) Performance plot of the trained network (b) training state and gradient analysis.

Now we can design a simple pattern recognition shallow neural network to classify the input image into different classes. Pattern recognition networks are feedforward networks that can be trained to classify inputs according to target classes. The target data for pattern recognition networks should consist of vectors of all zero values except for a 1 in element “I,” where “I” is the class they are to represent. Preparing the training dataset based on our very complete database is very easy. The input vector for the classifier is the (in this research 1 × 384 sized) eigenvalues vector, the output of SVD. The row vector of two hidden layer sizes 10 neurons. For the training function scaled conjugate gradient backpropagation is selected and the performance function is cross entropy. Figure 7 shows the network topology and Fig. 8 shows the training state and performance of the trained network. From the performance of 0.2 we can see that the training is done well and successfully. Hence we can state that using size reduced frequency transform as the feature map, we can introduce a new method of feature extraction based on biomedical resonance theory.

### 4.2 Method evaluation and comparison

Nowadays, in biomedical image processing, for semantic segmentation and image classification purposes, deep and shallow neural networks are used.

Deep networks such as U‐Net^26^ are very precise and they include filtering, image processing, feature selection, feature extraction, and classification in the network topology. However, training the network is very hard and needs resources. It may take days to label the whole database for preparing the ground truth for the network. Moreover, it would also take days to train the network with such a large dataset, and one training does not guarantee the perfectness of the network performance. Furthermore, the computational complexity for each decision‐making and simulation is also very high. So it may not be very cost‐effective to use such a network, particularly if we can find a better solution.

**Figure 9:**
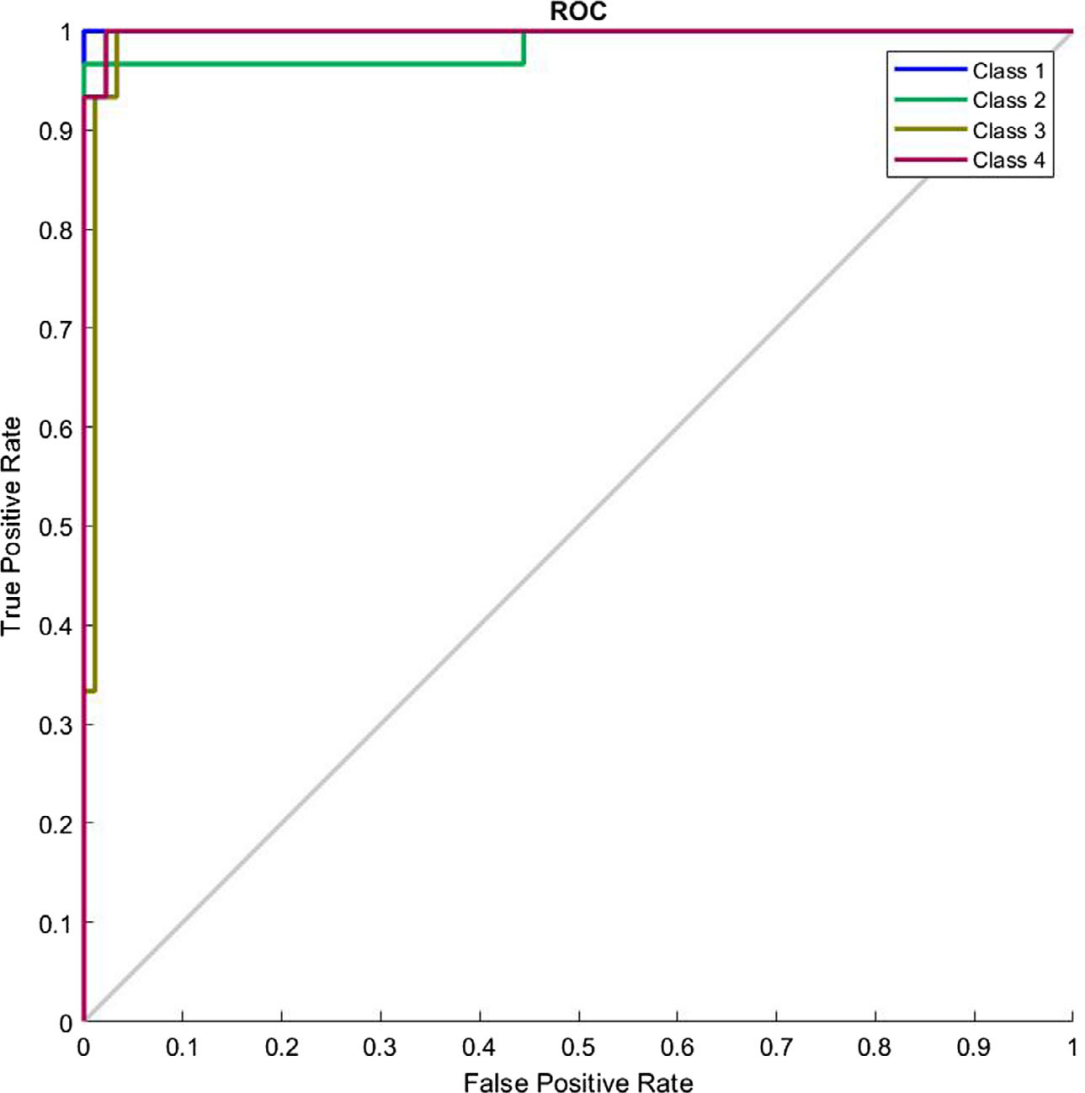
Receiver operating characteristic curve of the proposed classifier.

**Figure 10:**
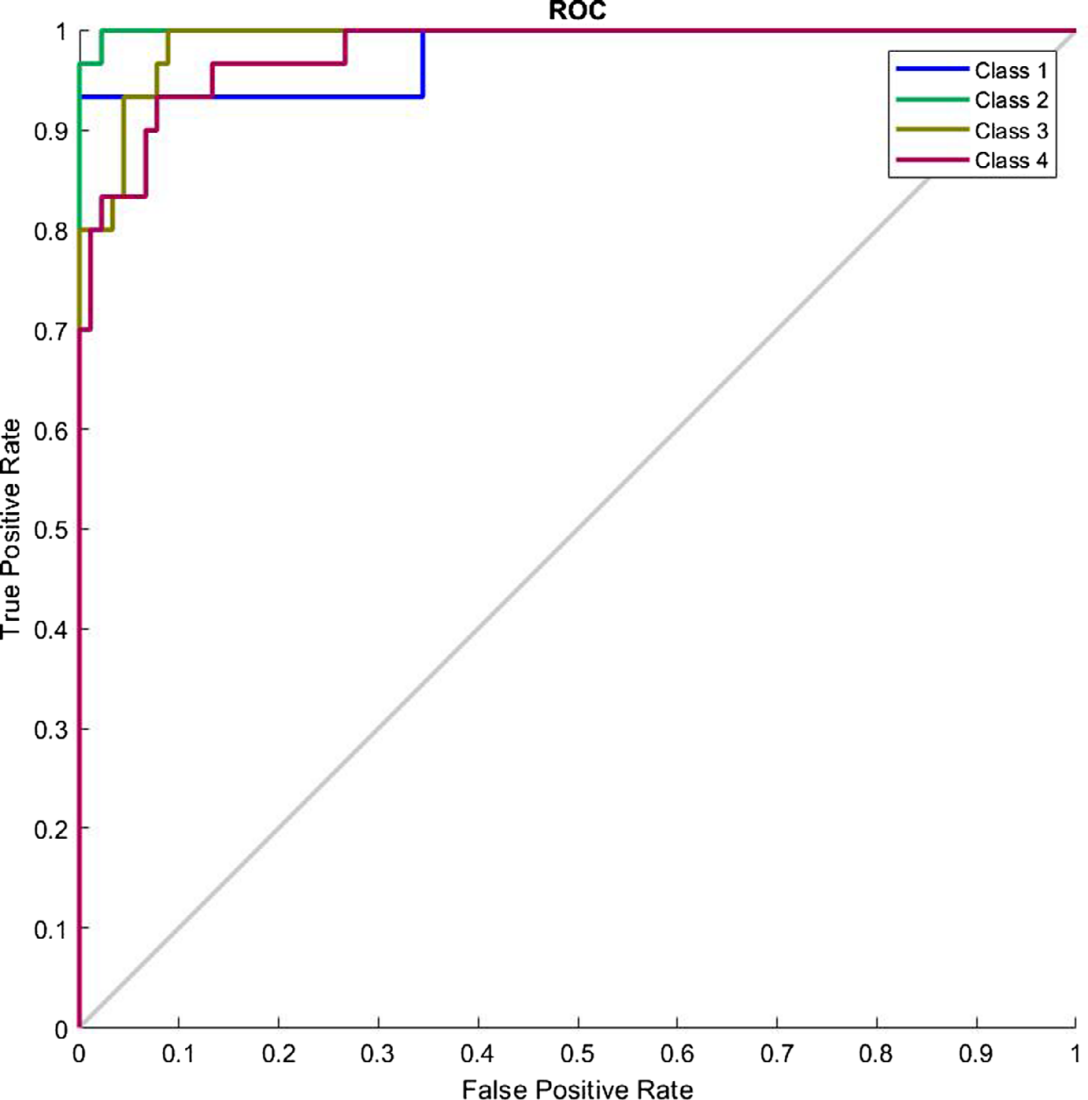
Plot classification confusion matrix of the proposed classifier.

**Figure 11:**
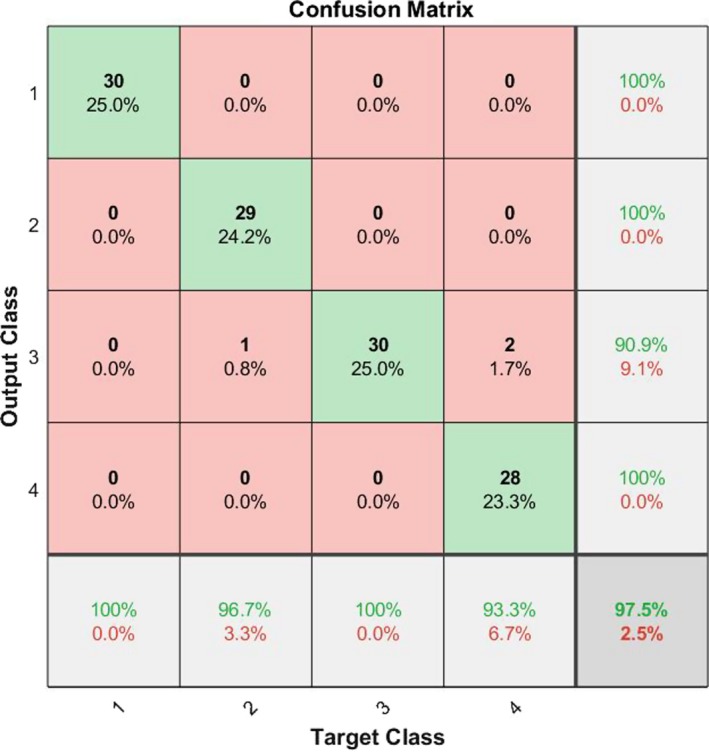
Receiver operating characteristic curve of the classifier presented in Ref. [2].

**Figure 12:**
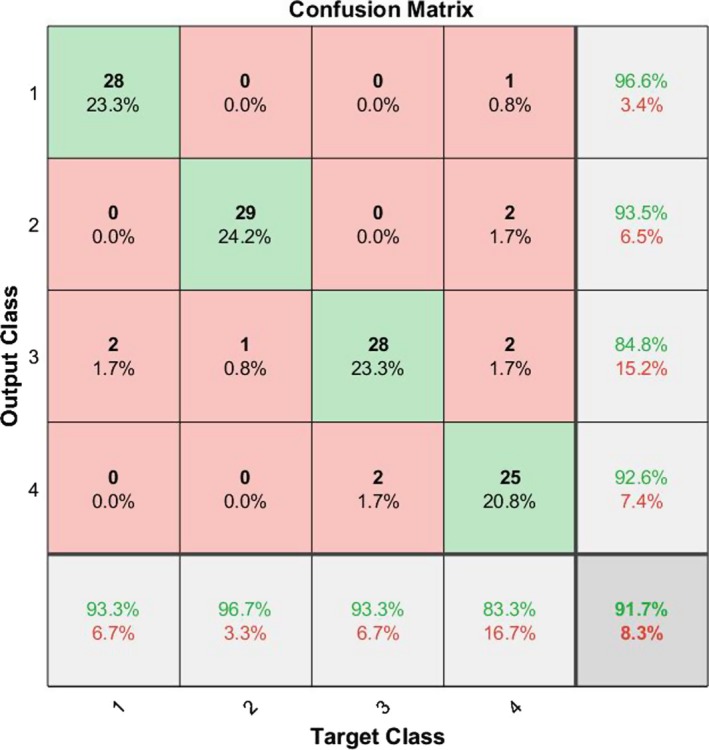
Plot classification confusion matrix of the classifier presented in Ref. [2].

In our last research on this database^2^ we presented an image processing procedure to extract the legion part if there exists any. Then we introduced some features and trained a complex multilayer perceptron neural network to classify the images. However we had two main troubles back then: (a) we needed an image processing algorithm to first find the lesion and (b) we had to select with which features we needed to train our network. In this research we have bypassed the need for the whole image processing and filtering part of the algorithm and have extended the feature size map from 4 to 384. Additionally we have also shorten the computation complexity and increased the diagnosis speed. This allows us for example to create a website able of processing the uploaded pictures by users all around the globe in the minimum needed time. Also in our last work we showed that the type of the cancer does not depend on the morphological shape of the lesion, instead it depends mostly on the statistical parameters of the lesion such as entropy and variance. This also enforces the idea of bioresonance theory presented in this research. Figure 9 shows the ROC curve of the classifier presenting the good diagnosing result. Figure 10 shows the confusion matrix and Table 2 presents the percentages values (represents the percentage of false negatives, false positives, true positives, and true negatives) for the proposed algorithm. Figure 11 shows the ROC curve of the proposed algorithm in Ref. [2] and Table 3 and Fig. 12 show the confusion values. From these evidences we can see the improvements of the new classification based on frequency features concluded from bioresonance theory. The reader must note that we have used a very simple pattern recognition classifier, for better results more hidden layers with more neurons and diverse activation functions can be used in the classifier.

**Table 2:** Confusion table of the proposed approach.

Classes	FN	FP	TP	TN
Class 1	0	0	100	100
Class 2	1.1	0	100	98.90
Class 3	0	9.09	90.91	100
Class 4	2.17	0	100	97.83

**Table 3:** Confusion table of the approach presented in Ref. [2

Classes	FN	FP	TP	TN
Class 1	2.20	3.45	96.55	97.8
Class 2	1.12	6.45	93.55	98.88
Class 3	2.30	15.15	84.85	97.7
Class 4	5.38	7.41	92.59	94.62

### 4.3 Sonogram semantic segmentation

**Figure 13:**
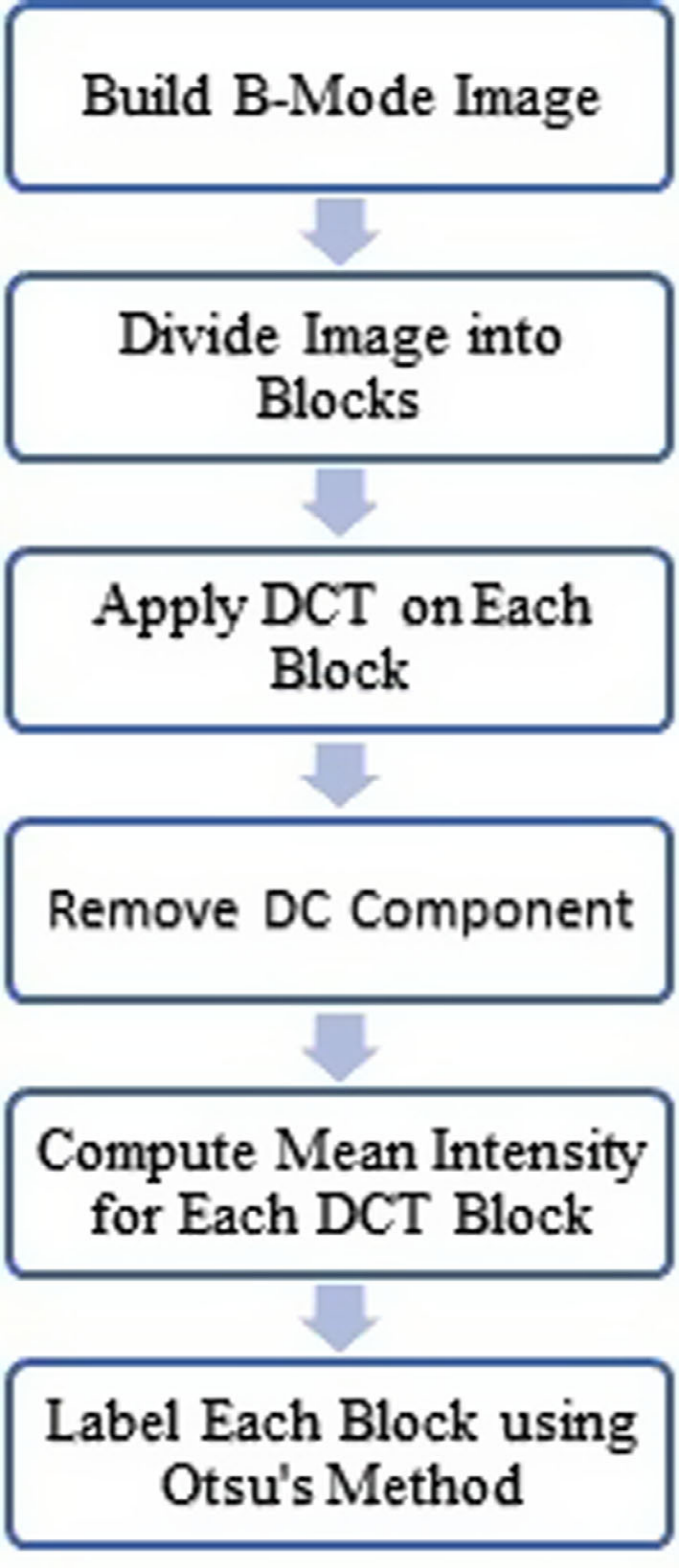
Procedure scheme for sonogram sematic segmentation.

The second method is used for sematic segmentation of the sonogram in order to differentiate the healthy parts from the suspected parts. The flowchart of this method is presented in Fig. 13 and the algorithm is described below.

#### 4.3.1 Dividing sonogram into blocks

The first step is to split the image. On one hand, the image frequency is a cumulative concept, and there is no frequency value for a single pixel; on the other hand, if the frequency transform is applied to the whole image, there will be a common frequency result in all scanned sectors. To be more specific, we can divide the image into blocks, and apply the frequency transform to each, and eventually reconstruct the image using the resulting blocks. An important consideration here is the dimensions of each block.

**Figure 14:**
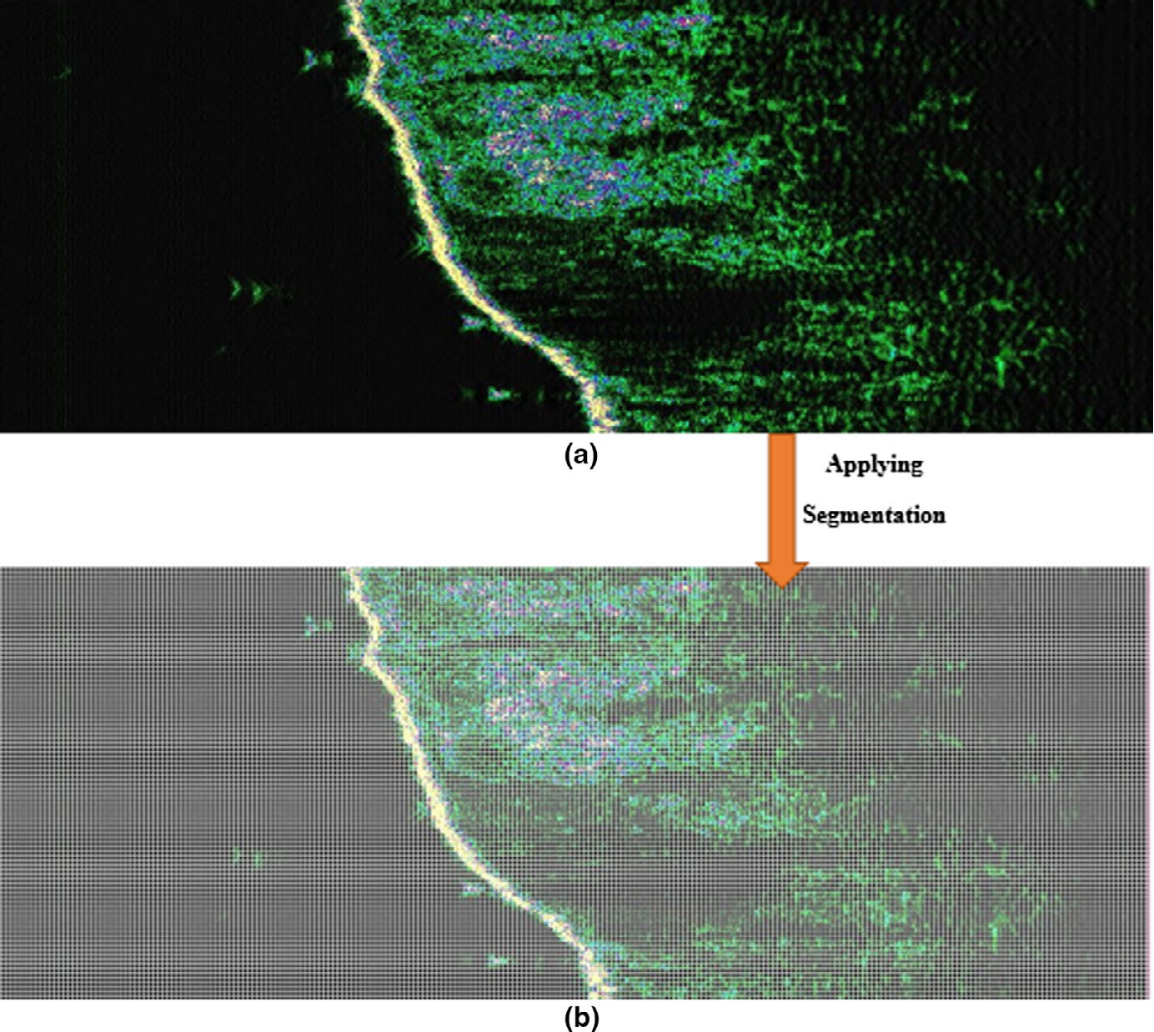
Cropped and segmented image with 4x4 resolution (a) original B‐mode (b) segmented.

If the frequency conversion is applied to the entire image, the result of the diagnosis is obtained, but if the process is performed for each block, it can be determined which part of the tissue is still healthy or damaged precisely. The whole image is split into blocks that block size plays an important role in the accuracy of analyzes. The minimum size for each block is 4 × 4 pixels, but in sonogram with a resolution of 1024 × 384 (as in this research), 32 × 32, and 16 × 16 blocks are optimal blocks that contain neighboring frequencies in comparison to the exact detection of each image. Figure 14 shows the properly segmented and cropped image.

#### 4.3.2 Apply DCT transform on each block

Two main notes are important here; first, for the detailed analysis, there is a need for alternations in the textual data, so the DC portion of the transformation should be eliminated, and secondly, there is only a need for the transform amplitude index. The DCT matrix of each block is as large as the original block in the primary image.

#### 4.3.3 Feature extraction and segmentation

After using DCT conversion and DC removal, a two‐dimensional matrix is calculated for each block, which represents the average intensity of each frequency in the block. Each row and column of this resulting matrix actually represents a frequency of the same block, and each element of that is in fact derived from the correlation of the image signal with that specific and constant frequency. Thus, by performing a frequency conversion, a matrix will be obtained, in which the elements indicate how many pixels of each block have that row frequency and that particular column frequency. More precisely, the elements of the resulting matrix indicate the intensity of the presence of that specific frequency in that block.

According to researches and studies related to this project, in general, healthier parts of the tissue have a higher average value for the frequencies in the image.^13^ However, the intensity of different frequencies in a particular sonogram is a relative property that is not absolute. Therefore, judging whether it is high or low should be taken separately in each image and based on its characteristic parameters. In the sense that it cannot be expressly stated that, for example, if the frequency value in images or in blocks was less than a constant limit, it indicates the malignancy of the lesion in the image captured in that photograph or not. Accordingly, they must be individually calculated for each block in accordance with their own parameters, and then accurate values for the threshold of the frequencies should be calculated and determined as the main golden feature used in the diagnosis process. This is due to the complexity of the information in the signals and some of the inherent differences in the skin of different people. This causes complexity and difficulty as well as the sensitivity of the diagnostic process. To achieve a high‐power precision method, many calculations and surveys were carried out.

Finally, the Otsu's adaptive method is selected to perform auto‐cluster‐based image thresholds^27^, ^28^ based on investigations. This is completely as the solution here matches. This criterion gives judgment for each part of sonogram in comparison with other parts. Ultimately, the ability to separate any sonogram into suspicious and healthy blocks will be created by doing the above calculations and comparing with the thresholds.

In image processing, Otsu's method, is used to automatically perform clustering‐based image thresholding^15^ or, the reduction of a gray level image to a binary image. The algorithm assumes that the image contains two classes of pixels following bimodal histogram (foreground pixels and background pixels), it then calculates the optimum threshold separating the two classes so that their combined spread (intraclass variance) is minimal, or equivalently (because the sum of pairwise squared distances is constant), so that their inter‐class variance is maximal.^28^

In this way, the matrix obtained in the previous step, which is the frequency matrix, enters the calculation program, and the output of the program will be a number, which will be the threshold for that image. Therefore, by applying this program for each image, the threshold for determining the healthy blocks from the lesion blocks of that image is determined by the intensity of the presence of different frequencies in each block of that image.

When the threshold value is found for each image, the image is reset to the original matrix and the following steps are reexamined. From the original image of the RF, a B‐mode image will be created and then the image will be blocked. In the next step, for each block, the DCT conversion is taken and the average value of the frequency for that block is calculated. Then this average value is compared with the frequency threshold of this image. Now, if the average frequency of that block is higher than the threshold of the frequency of this image, according to the concepts, it can be concluded that this block is healthy and there is no need for future research. It does not include a lesion, so you can mark this block as a healthy block and proceed to analyze the next block.

### 4.4 Computation results

**Figure 15:**
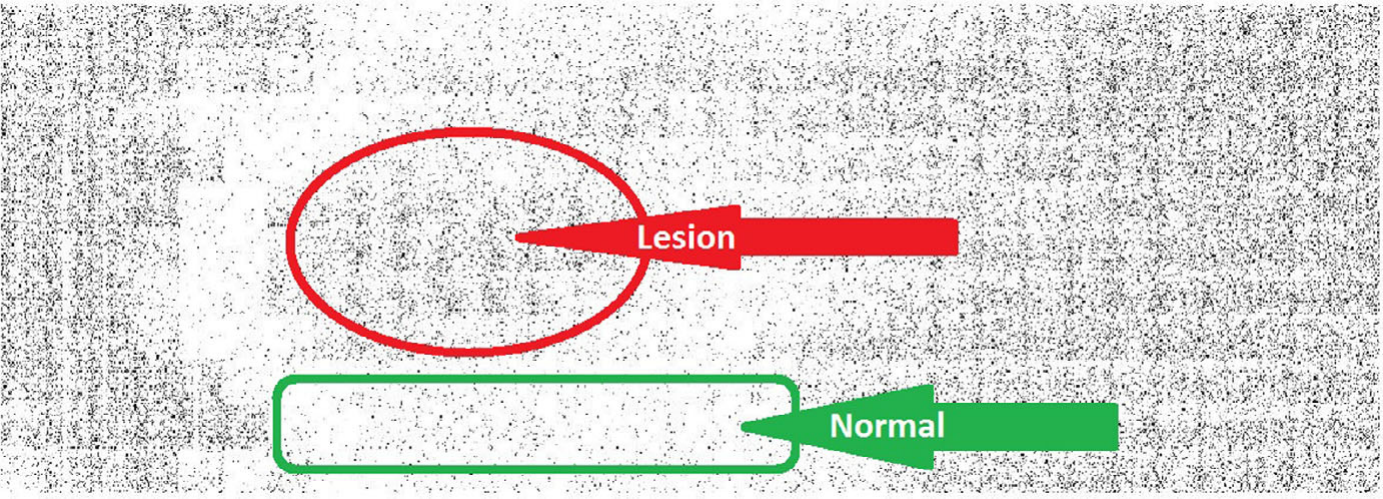
Reassembled image after applying frequency transform on the blocks to show the health or disease status of different parts of the skin tissue.

According to Fig. 5, if we apply DCT transformation to image blocks and combine the whole image using the resulting frequency matrices, a reconstructed image (Fig. 15) is obtained that shows the frequency characteristics of each block.

**Figure 16:**
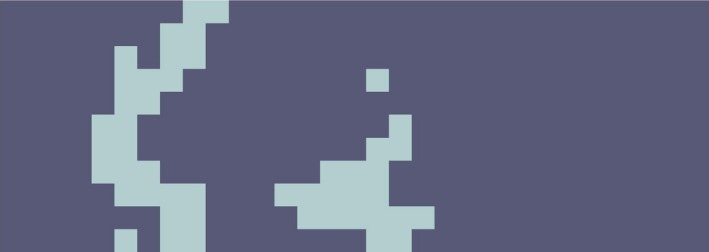
Final classification of each block according to the selected threshold.

Now, using the threshold of the frequency of each block calculated by the Otsu's method and by comparing it with the average frequency of each block, the ability to judge the condition of that part of the skin is created. To provide a detailed and easy image analysis for a user or expert, each block of an image can be represented in a color that expresses its status. In the constructed picture shown in Fig. 16, the lesion parts of skin are shown in dark color and healthy parts in bright color.

**Figure 17:**
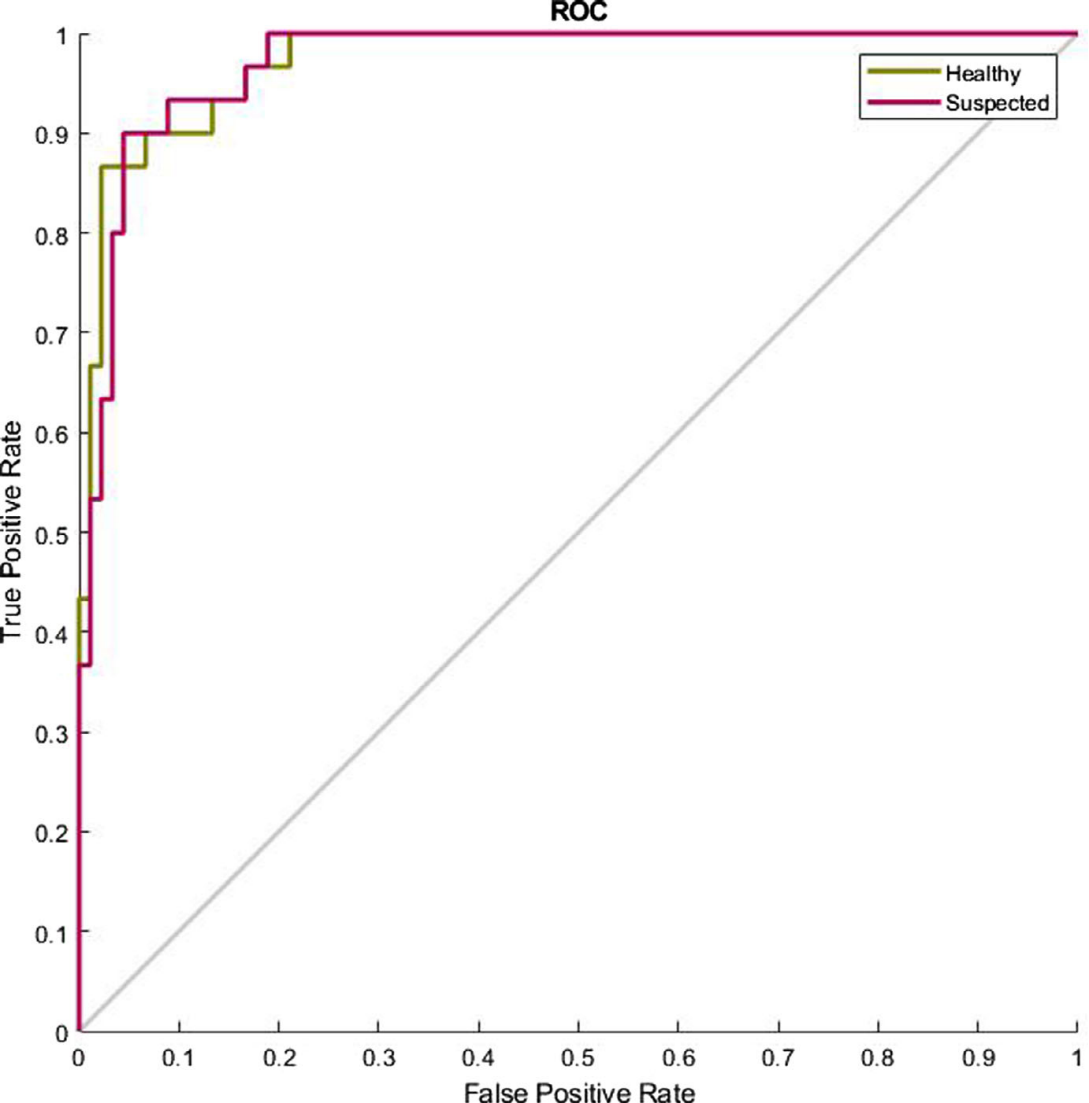
Receiver operating characteristic curve of the semantic segmentation.

The designed algorithm is evaluated with the mentioned database and the results are shown in Fig. 17. The region under receiver operating characteristic (ROC) curve is wide and indicates the quality of estimation. The research at the calculated threshold has been received AUC = 0.859, for sensitivities 93.8%, the specificities were 97.3%.

Since usually the sonograms' size is not very large (In this case 384 × 1024), considering the block size of 32 × 32 pixels, we will have a number of 384 blocks for each sonogram. Computing DCT for a 32 × 32 image does not take much time (in the range of milliseconds) and so dividing the input image into blocks and applying DCT on each image will take 2 s most. Computing mean for each DCT block takes microseconds, and since Otsu's method for 384 elements is very fast, the whole procedure may take seconds for a low‐speed computer.

In other words, this noninvasive approach is highly precise, while it is quick and cost‐effective to detect skin lesions malignant and reduces rate of false negative and false positive estimates that lead to additional costs and psychological stress on suspected patients.

## 5 CONCLUSION

As mentioned in the previous sections, a lot of research has been done to develop new methods for diagnosis of skin diseases. In most of them, there has been a great effort to find and develop a noninvasive detection method with high accuracy during an intelligent procedure, some of which have been mentioned in the previous sections and referred to in the references. In each of these studies, several indicators have been considered for the examination of the skin malignancy: for example, chemical vibrational modes of molecules within tissue,^32^ terahertz time‐domain spectroscopy for analyzing the thickness of tissue as a classification index,^33^ reflectance confocal microscopy (RCM),^34^ electrical impedance spectroscopy,^35^ optical coherence tomography,^36^ and some other methods have used different index parameters for skin disease diagnosis.

In this research, a novel and innovative way to achieve the ability to diagnose skin malignancy is presented. Based on this research concepts, the biological organism has a cellular oscillations, whose frequencies depend on the type of cells in that part of tissue or organ, where there is a specific frequency for a specific tissue under normal conditions, and if the tissue conditions change and there is a complication, then its natural frequency will be changed. So that the frequency response of these two parts and, consequently, the frequency of the return echoes received by the ultrasound probe, are different from each other. This difference is the basis of the classification in this study. As is detailed in the text of the article and in the previous sections, the innovation and novelty of this study is to use skin tissues natural frequency variations as a very suitable and powerful bio marker of skin malignancy for the diagnosis of skin tissue conditions. Moreover, an important point, and another aspect of being innovative, is to use ultrasound returned frequency analysis to find out the frequency characteristics of the tissue.

The presented algorithms (both classification and sematic segmentation) are all implemented in MATLAB R2018b using a laptop computer with quad core 2.8 GHz speed Core‐i7 processor and 16 GB of RAM. For the classification part of the research, creating a feature dataset for training the neural network, using feature extraction based on SVD dimension reduction of DCT matrices, for a database of 300 sonograms and training the pattern recognition network takes at most 1 min using the mention hardware specification. However, for the proposed algorithm presented in Ref. [2], creating features dataset and training the network from a database of 120 sonograms takes around 15 min. The diagnosis procedure takes <500 ms, meanwhile the proposed approach in Ref. [2] takes at least 3 s, depending on how the image processing is implemented. This issue becomes a trouble when one needs to implement the diagnosis algorithm as a web service, also intends to make the neural network adaptable. Hardware resources of servers are expensive and considerable. Moreover, for the segmentation part, the presented segmentation algorithm needs no networks to be trained, comparing to common intelligent methods of segmentation like U‐Net. The segmentation procedure of a 384 × 1024 sonogram using 32 × 32pixels blocks takes around 0.1 s for the mentioned hardware, while simulating a U‐Net network with such an image would take much longer; not to mention that training a deep network with a database containing only 20 images would take about 30 min on such system with a NVIDIA GTX1050 GPU. An adaptive deep network on a web server for such purpose would require a lot of resources. Overall, the proposed algorithms for classification and segmentation do not need an advanced hardware and could be easily executed in less than a second on an affordable 1.8 GHz Core‐i3 CPU with an 8 GB of RAM (recommended hardware).

Using these ideas and concepts to diagnose skin condition has created a very high level of fast, noninvasive and accurate detection that is evaluated by experimental database and could help dermatologists, to improve the accuracy of skin cancers diagnosis via a noninvasive and real‐time approach.

## CONFLICT OF INTEREST

None declared.
